# Effect of fresh frozen plasma on the in vitro activation of U937 monocytes: a potential role for the age of blood donors and their underlying cytokine profile

**DOI:** 10.1186/s40659-017-0146-3

**Published:** 2017-12-21

**Authors:** Mariana Patlán, Fausto Sánchez-Muñoz, Luis M. Amezcua-Guerra, Adriana Granados, Araceli Páez, Felipe Massó, Ana M. Mejía, Angeles Soster, Rafael Bojalil, Lenin Pavón, Luis A. Jiménez-Zamudio, Ricardo Márquez-Velasco

**Affiliations:** 1 Doctorado en Ciencias Quimicobiológicas, Escuela Nacional de Ciencias Biológicas, Instituto Politécnico Nacional, Mexico City, Mexico; 2 Department of Immunology, Instituto Nacional de Cardiología Ignacio Chávez, Juan Badiano No1, Col Sección XVI, 14080 Mexico City, Mexico; 3 Department of Health Care, Universidad Autónoma Metropolitana-Xochimilco, Mexico City, Mexico; 4 Department of Physiology, Instituto Nacional de Cardiología Ignacio Chávez, Mexico City, Mexico; 5 Blood Bank, Instituto Nacional de Cardiología Ignacio Chávez, Mexico City, Mexico; 6 Neuropsicoimmunology Laboratory, Instituto Nacional de Psiquiatría Ramón de la Fuente, Mexico City, Mexico; 7 Department of Immunology, Escuela Nacional de Ciencias Biológicas, Instituto Politecnico Nacional, Prolongación de Carpio y Plan de Ayala s/n, Santo Tomas, 11340 Mexico City, Mexico

**Keywords:** Fresh frozen plasma, Monocytes, Cytokines, Inflammation

## Abstract

**Background:**

Fresh frozen plasma (FFP) administration may increase the risk of nosocomial infections in parallel with the development of immune modulation. This could be driven by soluble mediators, possibly influencing the in vitro activation of human U937 monocyte cells, in a manner dependent on the age of the donors.

**Methods:**

FFP donors were stratified into groups of 19–30 years, 31–40 years or 41–50 years, and U937 cells were cultured with FFP (alone or plus lipopolysaccharide—LPS) for 24 h. Both in FFP and supernatants, TNF, IL-1β, IL-6, and IL-10 levels were measured by ELISA. Additionally, *CD11B*, *TLR2*, and *CASP3* gene expression were measured by qtPCR in U937 cells. Total phagocytic activity was also assayed.

**Results:**

Elevated IL-10, but low TNF and IL-1β levels were measured in FFP from individuals aged 19–40 years, whereas in individuals aged 41–50 years FFP were characterized by equalized TNF and IL-10 levels. Elevated IL-6 levels were found in all FFP samples, especially in those from the oldest individuals. FFP stimulation was associated with striking modifications in cytokine production in an age-dependent way. Exposure to FFP attenuates the response to LPS. *TLR2* and *CD11B* expression were enhanced regardless of the age of plasma donors, although *CASP3* expression was increased only when FFP from individuals aged 19–40 years were tested. Phagocytosis decreased after exposure to FFP regardless of donor age.

**Conclusion:**

Our results suggest that soluble mediators in FFP may modulate the functioning of monocytes. Interestingly, this effect appears to be partially influenced by the age of donors.

## Background

Fresh frozen plasma (FFP) is the fluid portion obtained from one unit of blood, which is processed by centrifugation and frozen at − 18 °C until used. It contains many soluble molecules such as clotting factors, fibrinogen, albumin, electrolytes, and natural anticoagulants [1, 2]. The use of FFP as adjunct therapy is common in different clinical settings. For instance, more than 4,000,000 units of FFP are transfused in the United States each year [3], and approximately 30% of patients in intensive care units will receive FFP transfusion at any point during their hospitalization [4, 5]. However, transfusion of FFP and other hemoderivatives has also been associated with abnormalities in the inflammatory response [6–8], thus increasing the risk of infections and other adverse outcomes [9, 10]. A few in vitro models such as co-cultures of monocytes with packed red blood cells [11] and co-incubation with FFP plus lipopolysaccharide (LPS) have been consistent to demonstrate immune disturbances characterized by decreased release of tumor necrosis factor (TNF) in parallel with increased interleukin (IL)-10 levels [12]. Although the underlying mechanisms are poorly understood, it is possible that soluble mediators such as cytokines in blood derivatives may modulate the functioning of monocytes and other leukocytes [13–17]. In support, development of post-transfusion side effects, including anaphylaxis, hemolytic reactions, and transfusion-related acute lung injury (TRALI) has been associated with the presence of cytokines in FFP and other blood derivatives [18–21]. Furthermore, these mediators are typically found in sera from healthy individuals and these may be also identified in leukocyte-reduced blood derivatives including FFP [14, 22, 23].

In the notion that both type and level of circulating cytokines may vary according to age [24–26], we sought to explore the effect of FFP from age-stratified healthy blood donors on the in vitro activation of human cells from monocytic lineage.

## Methods

### Plasma samples

FFP from healthy donors were obtained by single-step heavy spin centrifugation (5000*g* for 5–7 min) in the blood bank at the *Instituto Nacional de Cardiología Ignacio Chávez* in Mexico City, Mexico [27]. Only FFP samples from male individuals aged 18–50 years old, weighing > 50 kg, and fasting for more than 6 h were included. Blood samples fulfilled all the scrutiny tests required by the local regulation [28]. Accordingly, individuals with hepatitis B or C viruses, human immunodeficiency virus, herpes, pneumonia, diabetes, coronary artery disease, neoplasm, blood diseases, diarrhea or acute respiratory disease, syphilis, gonorrhea, malaria, drugs use, and users of acupuncture or with a history of tattoos are excluded as potential donors.

Ten mL of FFP were obtained during the routinely collection and processing in the blood bank. Samples were processed in free-pathogen conditions and stored in aliquots at − 70 °C until used. For analysis, FFP samples were grouped according to the age of donors as follows: 19–30 years old (n = 11), 31–40 years old (n = 9), and 41–50 years old (n = 4).

The protocol was approved by the local ethics committee and study was performed in line with the Declaration of Helsinki and local regulations.

### Biochemical analyses

TNF, IL-1β, IL-6 and IL-10 levels were measured in FFP samples and supernatant of cultured cells by enzyme-linked immunosorbent assay (ELISA) using commercial kits according to the manufacturer’s instructions (BioLegend, San Diego, CA, USA).

### Cell cultures

The human monocyte U937 cell line was obtained from ATCC (Cat. #CRL-1593.2) and cultured in RPMI-1640 with sodium bicarbonate and HEPES (Sigma, Palo Alto, CA, USA), supplemented with 1% l-glutamine (Sigma), 10% heat-inactivated fetal bovine serum (Gibco-Invitrogen, CA, USA), and 1% streptomycin-penicillin (Gibco-Invitrogen). A total of 7.5 × 10^5^ U937 cells in a final volume of 2 mL/well containing 1000 IU of heparin/well as anticoagulant were placed in 24-well plates (Nunc, Denmark), incubated at 37 °C, 5% CO_2_, and cultured as follows: (1) RPMI medium only (negative control); (2) RPMI medium plus 1 µg/mL of *E. coli* serotype 055: B5 LPS (positive control); (3) RPMI medium plus 20% FFP; (4) RPMI medium plus 40% FFP; (5) RPMI medium with 20% FFP plus LPS; and (6) RPMI medium with 40% FFP plus LPS. Cultures were incubated for 24 h, supernatants were collected, centrifuged (3000 *rpm* at 4 °C), and stored in 1.5 mL aliquots at − 75 °C. Of note, each FFP was tested individually.

In addition, U937 cells were placed in vials with 750 µL Tripure Reagent (Roche Cat. 11667165001, Mannheim Germany) and frozen at − 75 °C until studied.

### Reverse transcription and polymerase chain reaction

To assess the expression of activation-related gene transcripts, RNA was extracted from U937 cells according to the manufacturer’s recommendations. Electrophoresis in 1% agarose gel was used to assess RNA integrity, and concentration and purity by absorbance 268/280 nm in a Nanodrop 100 (Thermo, USA). cDNA was generated using 250 ng of total RNA, random primers, and the Transcriptor first strand cDNA synthesis kit (Roche Cat. 04379012001, Mannheim Germany). Real time PCR was conducted with LNA hydrolysis probes from the Universal Probe Library Roche (UPL) (Roche, Cat 04683633001, Mannheim Germany), and intron spanning primers from Sigma. *TLR2* (NM_003264.3) F 5′-CGTTCTCTCAGGTGACTGCTC-3′, R‘3-TCTCCTTTGGATCCTGCTTG-’5, UPL probe 14; *CASP3* (NM_004346.3) F 5′-CTGGTTTTCGGTGGGTGT-3′, R 3′-CCACTGAGTTTTCAGTGTTCTCC-5′ UPL probe 34; *CD11B* (NM_000632.3) F 5′-GGCATCCGCAAAGTGGTA-3′, R 3′-GGATCTTAAAGGCATTCTTTCG-5′ UPL probe 9; and *GADPH* (NM_002046.3) F 5′-AGCCACATCGCTCAGACAC-3′, R 3′-GCCCAATACGACCAAATCC-5′ UPL probe 60.

One μL of each cDNA was amplified with 400 nM of primers, 100 nM of UPL probe, with the LightCycler TaqMan^®^ Master (Roche, Cat. 04535286001) followed by 45 cycles of 95° 10 s, 60° 60 s, and 72° 1 s. Reference gene *GAPDH* was used for relative quantification, according to the 2^−ΔΔCt^ method, using the non-stimulated cells as calibrator sample.

### Phagocytosis assays

To evaluate phagocytic activity, U937 cells (5 × 10^5^ cells/mL, in 24-well plates) were differentiated to macrophages by incubating with 2 ng/mL phorbol myristate acetate (PMA Cat P8139, Sigma) during 48 h. A phagocytosis assay kit (Cayman Chemical, USA) was performed according to the manufacturer’s instructions. Briefly, rabbit IgG FITC-latex beads solution was added in cell cultures (100 µL/mL), and these were incubated during 24 h at 37 °C, 5% CO_2_. Phagocytosis of FITC-labeled latex beads was assessed using a FACSCalibur (BD Bioscience, San Diego, CA, USA) flow cytometer.

### Statistical analysis

The results of cytokine levels and expression of molecules are expressed as mean  ±  standard error of mean, while phagocytosis activity is presented as percentage. Comparisons between groups were computed by the Kruskal–Wallis test (with multiple comparison post-test by Dunn). Analyses were 2-tailed, and a *P* <  0.05 value was set for significance.

The GraphPad Prism 4.02 software (GraphPad Inc, San Diego, CA, USA) was used for calculations.

## Results

Main demographic characteristics of blood donors are summarized in Table 1. As noted, characteristics such as weight, height, heart rate, diastolic and systolic blood pressure, temperature and serum levels of C-reactive protein were not significantly different among individuals when they were grouped by age.

**Table 1:** Main clinical characteristics of healthy blood donors

	19–30 years (n = 11)	31–40 years (n = 9)	41–50 years (n = 4)
Weight, Kg	75.3 ± 4.1	71.5 ± 2.4	80.9 ± 9.5
Height, cm	164.3 ± 2.4	168.4 ± 1.5	167.0 ± 2.1
Heart rate, bpm	69.5 ± 2.2	63.1 ± 1.7	68.5 ± 2.0
Systolic blood pressure, mm Hg	132.2 ± 3.8	126.0 ± 5.3	117.3 ± 4.6
Diastolic blood pressure, mm Hg	75.4 ± 3.9	71.1 ± 3.4	73.5 ± 4.2
C-reactive protein, mg/L	1.1 ± 0.4	1.0 ± 0.2	2.3 ± 1.2

All data are presented as mean ± standard deviation

### Cytokines in fresh frozen plasma

**Fig. 1:**
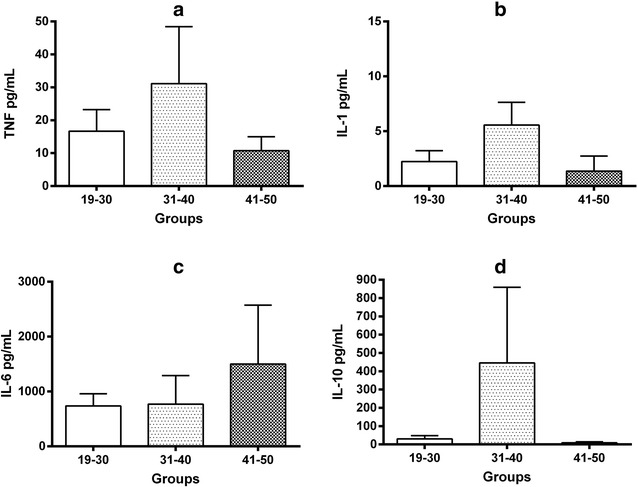
Cytokine levels in FFP. TNF, IL-1β, IL-10 and IL-6 ELISA measurements in FFP are represented in panels **a**–**d** respectively. Bars represent means and whiskers denote standard error. Measured were made for duplicate and the analyses between groups was made using a Kruskal–Wallis test with multiple comparison post-test by Dunn. Analyses were 2-tailed and a *P* <  0.05 value was used for significance

Cytokine levels were measured in FFP, and different inflammatory profiles were observed according to the age group. In donors aged 31–40 years, TNF concentration was 31.0 ± 17.3 pg/mL, IL-1β was 5.5 ± 2.0 pg/mL and IL-10 was 445.5 ± 413.2 pg/mL, whereas these figures were 10.7 ± 4.2, 1.3 ± 1.3, and 9.0 ± 5.2 pg/mL, respectively, in donors aged 41–50 years. Plasma of 19 to 30-year-old donors contained 16.6 ± 6.5 pg/mL of TNF, 2.2 ± 0.9 pg/mL of IL-1β, and 30.4 ± 17.8 pg/mL of IL-10 (Fig. 1, panels a, b, d). High IL-6 levels were detected in all FFP (19–30 years = 727.0 ± 222.0 pg/mL, 31–40 years = 768.0 ± 519.0 pg/mL, and 41–50 years = 1501.0 ± 1071.0 pg/mL) (Fig. 1, panel c).

### U937 cells stimulated with 20% fresh frozen plasma

**Fig. 2:**
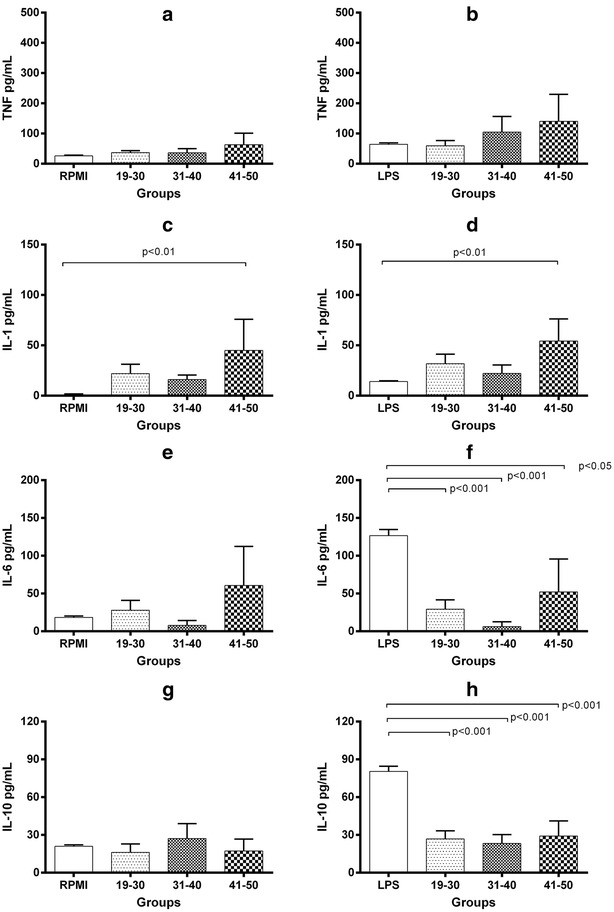
Cytokine levels in U937 cell cultures with 20% FFP. TNF, IL-1β, IL-10 and IL-6 were measured by ELISA in supernatants from cultured cells stimulated during 24 h solely with 20% FFP (panels **a**, **c**, **e**, **g**) or 20% FFP plus LPS (panels **b**, **d**, **f**, **h**). Assays were performed in triplicate. Bars represent means and whiskers denote standard error. Statistical analyses were performed using the Kruskal–Wallis test with multiple comparison post-test by Dunn. Analyses were 2-tailed, and a *P* <  0.05 value was used for significance

TNF levels in the supernatant of unstimulated U937 cells were 28.2 ± 1.9 pg/mL, while the exposure to 20% FFP induced levels of 36.7 ± 6.9, 36.5 ± 13.5 and 63.1 ± 38.4 pg/mL in 19–30 years, 31–40 years and 41–50 years groups, respectively (Fig. 2, panel a). Stimulation with LPS increased TNF production (64.1 ± 5.2 pg/mL) while co-stimulation with 20% FFP plus LPS induced age-dependent increments (19–30 years = 59.6 ± 17.1, 31–40 years = 104.8 ± 51.9, 41–50 years = 140.8 ± 88.8 pg/mL; P = ns) (Fig. 2, panel b). In addition, 0.9 ± 0.9 pg/mL of IL-1β were found in unstimulated U937 cells, and these levels increased after stimulation with 20% FFP (19–30 years = 21.9 ± 9.3 pg/mL, 31–40 years = 16.0 ± 4.5 pg/mL) reaching the highest level with stimulation with FFP of 41–50 years (45.0 ± 30.9 pg/mL; P < 0.01) (Fig. 2, panel c). LPS stimulation increased levels of IL-1β (14.0 ± 0.6 pg/mL), but unexpectedly, co-stimulation with 20% FFP plus LPS did not modify IL-1β levels compared to cells stimulated with FFP alone (Fig. 2, panel d).

In unstimulated cultures, IL-6 level was 18.4 ± 1.8 pg/mL, and exposure to 20% FFP induced 27.9 ± 13.2, 7.8 ± 6.5 and 60.7 ± 51.6 pg/mL as challenged with FFP of 19–30, 31–40, and 41–50 years, respectively (Fig. 2, panel e). LPS stimulation induced a significant increase in the IL-6 levels (126.5 ± 8.0 pg/mL), while co-stimulation with FFP + LPS was associated with modifications in the IL-6 levels as follows: 19–30 years = 29.1 ± 12.5 pg/mL, 31–40 years = 6.3 ± 6.3 pg/mL, 41–50 years = 52.3 ± 43.4 pg/mL (P < 0.001 for all comparisons) (Fig. 2, panel f). Finally, 20.9 ± 1.2 pg/mL of IL-10 were detected in unstimulated cultures, and these were not significantly modified by exposure to 20% FFP (19–30 years = 16.1 ± 6.7 pg/mL, 31–40 years = 27.1 ± 11.8 pg/mL, 41–50 years = 17.3 ± 9.4 pg/mL) (Fig. 2, panel g). In contrast, stimulation with LPS significantly increased IL-10 levels (80.4 ± 4.0 pg/mL), to a much greater extent than observed in the LPS plus 20% FFP co-stimulation (19–30 years = 26.8 ± 6.1 pg/mL, 31–40 years = 23.2 ± 6.6 pg/mL, 41–50 years = 29.1 ± 10.4 pg/mL; P < 0.001 for all comparisons) (Fig. 2, panel h).

### U937 cells stimulated with 40% fresh frozen plasma

**Fig. 3:**
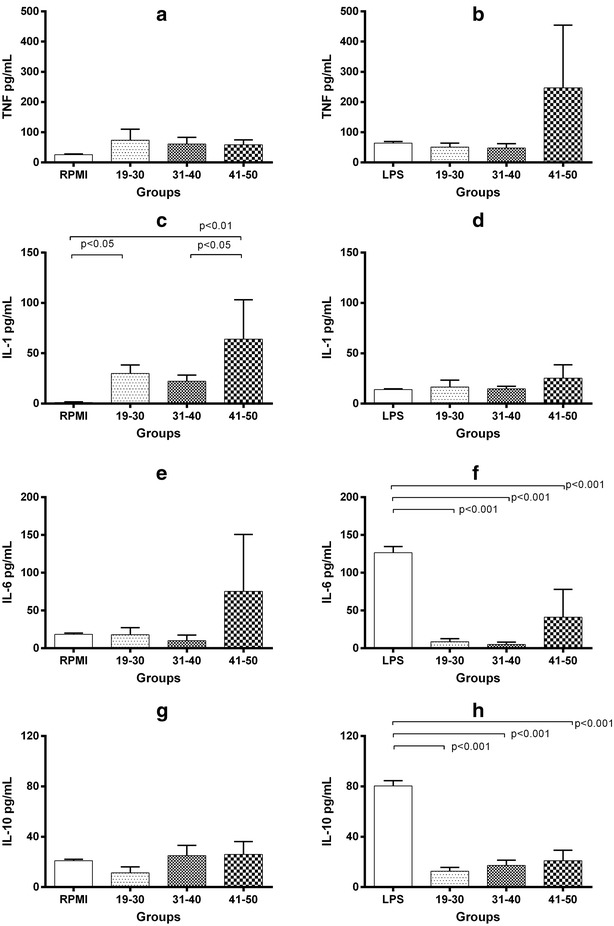
Cytokine levels in U937 cell cultures with 40% FFP. TNF, IL-1β, IL-10 and IL-6 were measured by ELISA in supernatants from cultured cells stimulated during 24 h solely with 40% FFP (panels **a**, **c**, **e**, **g**) or 40% FFP plus LPS (panels **b**, **d**, **f**, **h**). Assays were performed in triplicate. Bars represent means and whiskers denote standard error. Statistical analyses were performed using the Kruskal–Wallis test with multiple comparison post-test by Dunn. Analyses were 2-tailed, and a P <  0.05 value was used for significance

In U937 cells cultured with RPMI, 25.9 ± 2.0 pg/mL of TNF were measured, and stimulation with 40% FFP modified its levels to 74.1 ± 36.1 pg/mL in 19–30 years, 60.9 ± 2.1 pg/mL in 31–40 years, and 58.7 ± 15.9 pg/mL in 41–50 years (Fig. 3, panel a). Co-stimulation with 40% FFP plus LPS was no associated with important changes in TNF levels (19–30 years = 50.9 ± 13.2 pg/mL, 31–40 years = 47.7 ± 14.3 pg/mL, and 41–50 years = 246.7 ± 208.0 pg/mL (Fig. 3, panel b). Concerning to IL-1β, from a basal production of 0.9 ± 0.9 pg/mL, stimulation with 40% FFP induced significant increased levels in groups 19–30 years = 29.9 ± 8.4 pg/mL, and 41–50 = 64.0 ± 39.1 pg/mL, in the oldest group levels were higher than 31–40 years group (22.1 ± 6.0 pg/mL; P < 0.05) (Fig. 3, panel c). Although stimulation with LPS alone increased IL-1β levels to 14.0 ± 0.7 pg/mL, co-stimulation with LPS plus 40% FFP no longer modified them (19–30 years = 16.5 ± 6.5, 31–40 years = 14.6 ± 2.4, 41–50 years = 25.3 ± 11.5 pg/mL) (Fig. 3, panel d).

In unstimulated cultures, IL-6 level was 18.4 ± 1.8 pg/mL, and exposure to 40% FFP resulted in 17.91 ± 9.4, 10.0 ± 7.5 and 75.3 ± 75.3 pg/mL when co-cultured with FFP of 19–30, 31–40, and 41–50 years, respectively (Fig. 3, panel e). LPS stimulation increased IL-6 levels (126.5 ± 8.0 pg/mL, P < 0.001 in all comparisons with co-stimulated groups), while co-stimulation did not modify those levels observed with FFP alone (19–30 years = 8.8 ± 3.3, 31–40 years = 5.0 ± 3.1, and 41–50 years = 41.2 ± 36.5 pg/mL) (Fig. 3, panel f). Similar IL-10 levels were found in cells cultured with RPMI (20.9 ± 1.2 pg/mL) and in cells stimulated with 40% FFP (19–30 years = 11.3 ± 4.8 pg/mL, 31–40 years = 25.0 ± 8.1 pg/mL, 41–50 years = 25.9 ± 10.4 pg/mL) (Fig. 3, panel g). LPS stimulation alone increased IL-10 levels to 80.4 ± 4.0 pg/mL P < 0.001 for all comparisons), while co-stimulation with 40% FFP was associated to diminished IL-10 levels (19–30 years = 12.6 ± 3.0 pg/mL, 31–40 years = 17.2 ± 5.2 pg/mL, 41–50 years = 21.0 ± 8.3 pg/mL) (Fig. 3, panel h).

### Gene expression of activation molecules in U937 cells

**Fig. 4:**
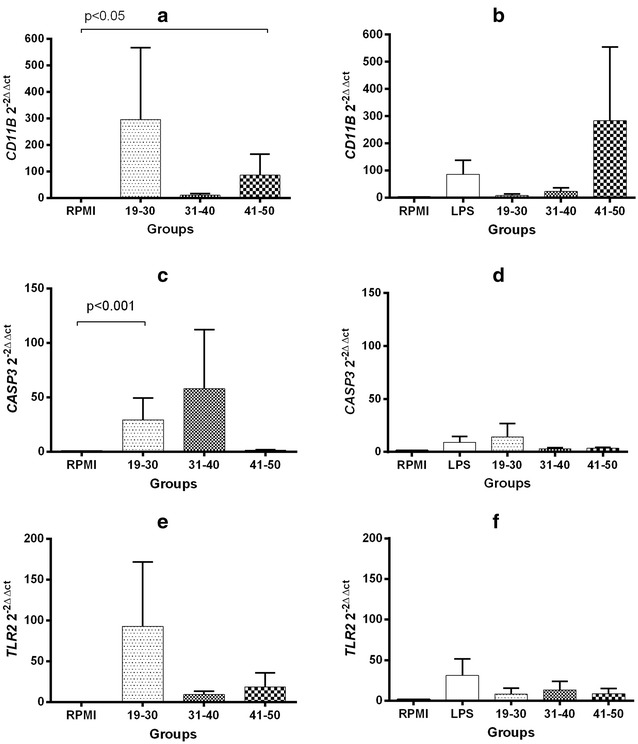
mRNA levels of activation-related molecules in U937 cells with 20% FFP. CD11b, Casp3 and TLR2 RT-qPCR measurements in cells stimulated 24 h solely with 20% FFP (panels **a**, **c**, **e**) or 20% FFP plus LPS (panels **b**, **d**, **f**). Bars represent means and whiskers denote standard error (mRNA levels according to the 2^−∆∆CT^ method). Assays were performed in triplicate. Statistical analyses were performed using the Kruskal–Wallis test with multiple comparison post-test by Dunn. Analyses were 2-tailed, and a P <  0.05 value was used for significance

The relative expression of molecules related to activation in U937 cells was normalized according to the levels of expression found in unstimulated U937 cells (RPMI). Exposure to 20% FFP of 19–30 years increased the expression of *CD11B* to 295.0 ± 270.0, whereas this increased to 10.3 ± 5.2 and 85.7 ± 80.1 (P < 0.05) when exposed to FFP of 31–40 and 41–50 years, respectively (Fig. 4, panel a). Stimulation with LPS induced an increase in the expression of *CD11B* (86.6 ± 51.3), while the co-stimulation with LPS plus 20% FFP of individuals of 19–30, 31–40 and 41–50 years resulted in 8.0 ± 5.9, 23.2 ± 13.4 and 283.2 ± 271.4 increased expression, respectively (Fig. 4, panel b). Exposure to 20% FFP of 19–30 years increased the expression of *CASP3* to 29.3 ± 19.9 (P < 0.001), whereas this increased to 58.0 ± 54.1, 1.3 ± 0.4 when exposed to FFP of 31–40 and 41–50 years, respectively (Fig. 4, panel c). Although stimulation with LPS increased the expression of *CASP3* (9.2 ± 5.3), co-stimulation with 20% FFP did not significantly modify this expression (19–30 years = 14.2 ± 12.6, 31–40 years = 2.9 ± 1.0, 41–50 years = 3.5 ± 0.9) (Fig. 4, panel d). The expression of *TLR2* increased in the presence of 20% FFP (19–30 years = 92.7 ± 78.7, 31–40 years = 9.1 ± 3.8, 41–50 years = 31.3 ± 20.3) (Fig. 4, panel e). However, while stimulation with LPS induced an increase in *TLR2* expression (31.3 ± 20.3), co-stimulation with LPS and 20% FFP was not associated with significant changes in *TLR2* expression (19–30 years = 8±7.5, 31–40 years = 13.3 ± 9.7, 41–50 years = 8.7 ± 6.3) (Fig. 4, panel f).

**Fig. 5:**
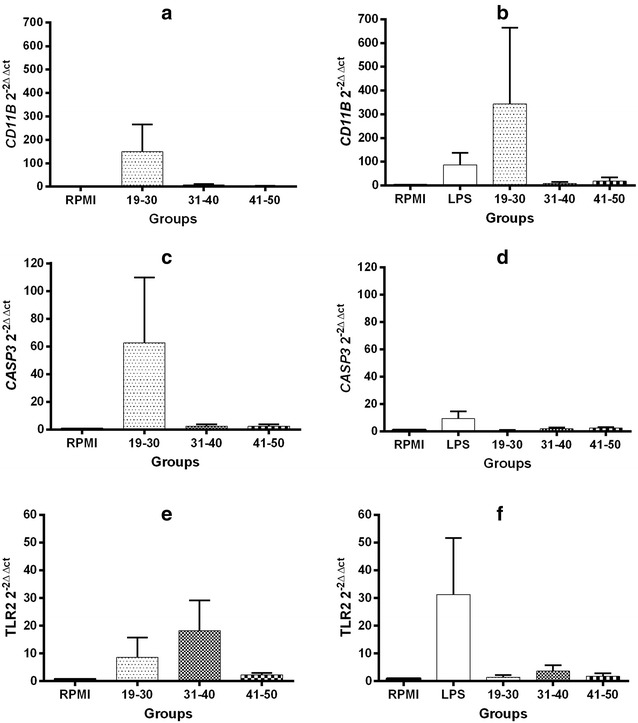
mRNA levels of activation-related molecules in U937 cells with 40% FFP. CD11b, Casp3 and TLR2 RT-qPCR measurements in cells stimulated 24 h solely with 40% FFP (panels **a**, **c**, **e**) or 40% FFP plus LPS (panels **b**, **d**, **f**). Bars represent means and whiskers denote standard error (mRNA levels according to the 2^−∆∆CT^ method). Assays were performed in triplicate. Statistical analyses were performed using the Kruskal–Wallis test with multiple comparison post-test by Dunn. Analyses were 2-tailed, and a P <  0.05 value was used for significance

As can be observed in Fig. 5 (panels a–f), the relative expression of any of the activation-related molecules was not significantly modified with exposure to 40% FFP of any of the age groups, either alone or in co-stimulation with LPS.

### Phagocytosis assays

**Fig. 6:**
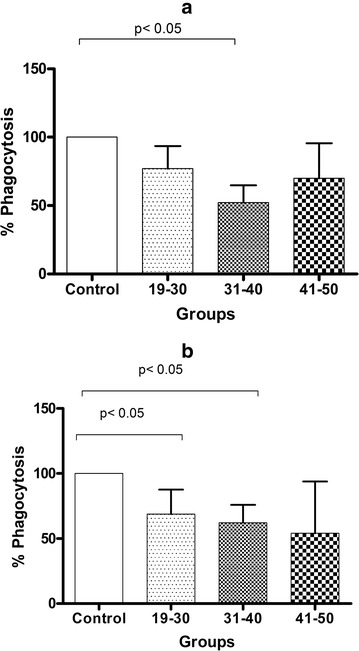
Phagocytosis assays in U937 monocytes with 20% FFP. PMA differentiated U937 cells stimulated during 24 h with 20% FFP (**a**) and 40% FFP (**b**). Phagocytosis assays were conducted using color-flow cytometry. Assays were performed in duplicate. Bars represent means and whiskers denote standard error. Statistical analyses were performed using the Kruskal–Wallis test with multiple comparison post-test by Dunn. Analyses were 2-tailed, and a P <  0.05 value was used for significance

Finally, total phagocytosis in U937 cells cultured with RPMI was used as 100% reference. The addition of 20% FFP was associated with a decrease in phagocytosis, although only the FFP of 31–40 years reached significance (19–30 years = 77% ± 16%, 31–40 years = 52.8% ± 12.6%, 41–50 years = 69.9% ± 25.5%) (Fig. 6, panel a). When phagocytic activity was evaluated in the presence of 40% FFP, a significant reduction was observed only in the 19–30 years (68.8% ± 18.8%; P < 0.05), and 31–40 years (62.1% ± 13.7%; P < 0.05) groups (Fig. 6, panel b).

## Discussion

This study assessed the effect of FFP from healthy donors grouped by age, on the in vitro activation of human cells from monocytic lineage. Here, we found that FFP exposure was associated with modifications in the production of inflammatory cytokines, the expression of activation-related genes, and in total phagocytosis. In addition, this study highlighted that age of blood donors as well as the type of cytokines present in plasma are relevant for both the magnitude and direction of inflammatory cell regulation.

Administration of FFP and other blood derivatives has been a cornerstone of the practice of medicine, although the development of serious side effects remains a major concern especially in critically ill patients [19, 21]. The presence of soluble mediators in blood derivatives may be causative of several of these side effects since they have the potential to modulate immune cells in the host [14]. In line with this, FFP exposure was associated with increased production of TNF and IL-1β, while precluded LPS-related activation and decreased phagocytosis in U937 cells. Therefore, our results suggest that FFP may act as an external source of cytokines, which in turn could induce transfusion-related, immune-mediated adverse events.

An important novelty of this study is the finding that the age of blood donors may have a role in the modulation of cell activation after FFP exposure. Indeed, a differential expression of cytokines in each age stratum might be responsible for individual cell responses in the host. An equalized TNF/IL-10 concentration in the presence of high IL-6 levels, as seen in FFP from 41 to 50 group, was associated with U937 cells activation and increased expression of *CD11B* and *TLR2*, even though a decreased phagocytic activity was observed. In contrast, higher IL-10 levels than TNF levels, as observed in FFP from 19 to 30 and 31 to 40 groups, was associated with decreased *CD11B* and *TLR2* expression but increased *CASP3* expression. Notably, the highest *CASP3* expression was found to be associated with decreased phagocytic activity. Despite endogenous TNF or IL-10 cannot explain the reduction in *CASP3* expression, the presence of high levels of IL-6 in FFP from individuals aged 41–50 could explain these results, at least partially. Indeed, IL-6 may suppress apoptosis through PI3-Kinase/Akt activation via the phosphorylation of BCL-2 family member BAD. Phosphorylated BAD is associated with 14-3-3 proteins which sequester BAD from BCL-X_L_, thereby promoting cell survival [29]. In addition, IL-6 may prevent *CASP3* overexpression by inhibiting IP3R-dependent Ca^2+^release-induced apoptosis [30]. Accordingly, FFP from donors aged 19–31 showed the lowest levels of IL-6 and these samples were responsible for the highest expression of *CASP3* as an apoptosis marker. This is further supported by the demonstration that IL-6 blockade may reduce tumor cell viability while promotes apoptosis [31]. Despite that stimulation with LPS significantly increased both cytokine production and expression of activation-related molecules, cells co-cultured with FFP presented only a slight increment in the TNF production as well as a decreased expression of activation molecules. Previously, Schneider and colleagues have shown that addition of a variety of blood derivatives such as PRBC or FFP significantly suppresses TNF production, especially in monocytes stimulated with LPS plus FFP [12]. The mechanisms underlying the inhibitory response associated with blood derivatives remain obscure, but a plausible explanation seems to involve the presence of soluble molecules in FFP. In fact, IL-1 receptor antagonist (IL-1RA) as well as the soluble TNF receptors (sTNFR) I and II are elevated in the serum of healthy individuals [32, 33]. These soluble receptors are known to bind directly to the free cytokine, thereby competing with membrane-bound receptors. The final effect of these soluble receptors may be the interference in cellular actions, altering the production of other cytokines. Alternatively, serum IgM and IgG anti-LPS antibodies have already been demonstrated in Balb/c mice treated with LPS, and anti-LPS antibodies with antibacterial activity were recently described in humans [34, 35].

Finally, even though FFP and other blood derivatives may approve the scrutiny of blood banks for infectious agents, they can bring cytokines and other soluble mediators reflecting inflammatory subclinical events occurring in otherwise healthy blood donors. Moreover, the normal physiological response that underlies any individual is changing during the course of life and it may be reflected in the cytokine levels according to the age of blood donors [14, 24, 25, 36].

We are aware that our study has several limitations. Firstly, we focused this work to assess several cytokines mainly found in early inflammatory responses, and the role that adaptive immune cytokines may play in monocyte activation remains to be elucidated. Secondly, we were unable to specifically inhibit each cytokine with specific anti-cytokine monoclonal antibodies due to technical difficulties, thus confirmation of a direct cause-effect relationship is still lacking. The role that age-related sex hormones conveyed in FFP may play in cell activation was not assessed. Finally, although FFP from a blood bank were used in this study, we must consider that other factors associated with donors’ lifestyle could influence the large differences observed in response to monocyte stimulation.

In conclusion, this study supports the notion that soluble mediators in FFP may modulate the activation and overall functioning of monocytes. In addition, our results suggest that these effects are related to the age of blood donors.

## Abbreviations

CASP: caspase
CD: cluster of differentiation molecules
cDNA: complementary DNA
ELISA: enzyme-linked immunosorbent assay
FFP: fresh frozen plasma
HEPES: (4-(2-hydroxyethyl)-1-piperazineethanesulfonic acid) buffer
IL-: interleukin
IP3: inositol triphosphate
LPS: lipopolysaccharide
PCR: polymerase chain reaction
PRBC: packed red blood cells
qPCR: real-time polymerase chain reaction
RNA: ribonucleic acid
RPMI: Roswell Park Memorial Institute medium
TLR: toll-like receptor
TNF: tumor necrosis factor
TRALI: transfusion-related acute lung injury
UPL: Universal Probe Library Roche
